# Empathy Mediates the Relationship Between Motivations After Transgression and Forgiveness

**DOI:** 10.3389/fpsyg.2020.01466

**Published:** 2020-07-08

**Authors:** Lin Ma, Yingjie Jiang

**Affiliations:** ^1^School of Psychology, Northeast Normal University, Changchun, China; ^2^School of Teacher Education, Anqing Normal University, Anqing, China

**Keywords:** revenge, avoidance, forgiveness, empathy, adolescents

## Abstract

Previous studies found the associations between motivations after transgression and forgiveness in adults. However, less is known about the relationship between transgression-related motivations and forgiveness among adolescents and the potential mediating role of empathy. These questions were investigated among 445 Chinese adolescents using the Tendency to Forgive Scale, the Transgression-Related Interpersonal Motivations Inventory, and the Interpersonal Reactivity Index. The results found a negative relationship between avoidance and revenge motivation and forgiveness tendency and a positive association between benevolent motivation and forgiveness tendency. In addition, the study also revealed a partial mediating role of empathy regarding the effect of the transgression-related motivations on forgiveness tendency. These findings suggested that empathy plays a vital role in the relationship between transgression-related motivations and forgiveness among adolescents.

## Introduction

People inevitably encounter conflicts and offense which will destroy interpersonal relationships in interpersonal interaction. Avoidance and revenge probably are two main motivations toward a perpetrator after being offended (McCullough and Hoyt, 2002). This is because negative emotional responses like fear, anger, or sadness are predominant responses toward offenses (Slotter et al., 2012; Civai, 2013; Gilam et al., 2019). However, people sometimes can restrain their instinctive impulses to retaliate or avoid and choose forgiveness as an effective strategy to maintain the relationship (Billingsley and Losin, 2017). Forgiveness is regarded as a changing process of pro-social behavior, which means that people give up revenge and avoid the offender and instead choose to show kindness to the offender (Enright et al., 1998; McCullough and Hoyt, 2002).

Previous studies on forgiveness mainly focus on adult samples (e.g., McCullough et al., 1997; McCullough, 2000) rather than on adolescents (e.g., Huang and Enright, 2000; Klatt and Enright, 2009; Barcaccia et al., 2017). Researchers proposed that the tendency to forgive increases as children grow up (Enright et al., 1989); 15- to 16-year-old adolescents would consider forgiveness under the pressure of other people, while adults would not (Enright, 1991). According to the defect mode of adolescent development, adolescence is a period of great turbulence during which they have to face great physical and mental changes and are vulnerable to the adverse effects of their peers (Roth et al., 1998). Therefore, after encountering conflicts, adolescents may adopt a maladaptive coping response to interpersonal offenses because of immature thought (Worthington and Scherer, 2004), which may lead to retaliation or avoidance that will damage the interpersonal relationship. However, a positive coping strategy like forgiveness may avoid the negative effects of conflict and remove the negative emotions (Yao and Enright, 2018).

Although there is a growing interest in the research of forgiveness among psychologists (e.g., McCullough et al., 1997; Worthington et al., 2001; McCullough and Hoyt, 2002; Toussaint et al., 2015), the definition of forgiveness is still inconsistent (Berry et al., 2005; Worthington, 2007; Riek and Mania, 2012). McCullough et al. (1997) believed that forgiveness is a pro-social change process of motivation, which inclines people to behave by constructive motivation in order to suppress destructive motivation. The tendency to forgive refers to one’s global dispositional level of forgiveness across contexts (Brown, 2003). Previous studies suggested that forgiveness can help individuals overcome interpersonal violations, especially negative emotions such as anger, worry, fear, and embarrassment, reduce individual anxiety and depression, and improve self-esteem, subjective well-being, and life satisfaction (e.g., Worthington and Wade, 1999; Maltby et al., 2001; Seybold et al., 2001; Bono and McCullough, 2006; Reed and Enright, 2006). The results associated with forgiveness and positive outcomes are found not only in adult studies (e.g., Maltby et al., 2001; Berry et al., 2005; Lawler-Row et al., 2011) but also in adolescent studies (e.g., Benda, 2002; Gambaro, 2003; Barcaccia et al., 2020). When exploring the role of forgiveness in the mental health of middle school adolescents, Gambaro (2003) found that forgiveness can significantly reduce their anger, decrease the adverse consequences of anger, and improve interpersonal relationships. Hui and Chau (2009) investigated the effect of forgiveness intervention within Chinese adolescents, and the findings revealed that process-based forgiveness interventions are effective for adolescents to improve their psychological well-being. Recent studies on forgiveness in the context of school bullying and Internet bullying found that forgiveness and friendship are protective factors for adolescents to avoid harm and reduce anger, while unforgiveness is significantly related to high depression and revenge (e.g., Barcaccia et al., 2017, 2018; Watson et al., 2017). Moreover, teenagers who suffer from Internet bullying report less internet violence if they have a higher tendency of forgiveness (Quintana-Orts and Rey, 2018).

So, how can we grant forgiveness or improve the tendency of forgiveness? Researchers proposed that empathy, a social cognitive factor, has a stable effect on forgiveness (e.g., McCullough et al., 1997, 1998; Worthington et al., 2001; McCullough and Hoyt, 2002; Zechmeister and Romero, 2002; Fourie et al., 2020). Empathy refers to the ability of sharing and understanding others’ emotion and feelings (Decety and Jackson, 2006). McCullough et al. (1997) found that studying empathy-based forgiveness courses is effective in promoting the participants’ forgiveness. According to Baston’s theory of empathy altruism, researchers speculated that empathy makes individuals care for the needs of the offender, perceive that the offender is also experiencing guilt and pain, and then hope to reconstruct a positive contact with the offender and then promote forgiveness (McCullough et al., 1997). The study also found that empathy has a direct impact on forgiveness and plays a mediating role on forgiveness and apology (McCullough et al., 1998). Some researchers explored the reasons why empathy promotes forgiveness and found that the victims of high dispositional empathy attribute the offense positively and are more likely to forgive the offender (Zechmeister and Romero, 2002). This important role of empathy was also found in studies of forgiveness among adolescents. Hui and Chau (2009) found that empathizing with the offender and thinking from the perspective of others is a key strategy in the process of forgiveness. Adolescents with higher levels of empathy report more frequent forgiveness in the face of relationship aggression than those with lower levels of empathy (Johnson et al., 2013).

Previous studies explored the relationship between motivations after aggression and forgiveness, and empathy for the offender will promote forgiveness toward the offender among adults). However, the association between transgression-related motivations, empathy, and forgiveness is still unclear among adolescents. Therefore, in the current study, we aim to examine the link between transgression-related motivations and forgiveness in adolescents and the potential mediating role of empathy. Based on previous studies (McCullough et al., 1997, 1998; Hui and Chau, 2009; Johnson et al., 2013; Barcaccia et al., 2018), we assumed that the motivations of revenge and avoidance after transgression was negatively correlated with empathy and forgiveness, while the benevolence motivation was positively correlated with empathy and forgiveness. In addition, empathy may mediate the relationship between transgression-related motivations and forgiveness.

## Methods

### Participants

Data were collected from 445 junior and senior high school students (188 males, 257 females) from China of ages 12–17 years (*M* = 15.51, SD = 1.47). The percentage of subjects with ages from 12 to 17 years was 3.4, 17.1, 1.3, 4.0, 51.5, and 22.7, respectively. All students were asked to complete paper-and-pencil questionnaires individually or in class groups. The participants were compensated for by a small gift after completing questionnaires. The study was approved by the Ethics Committee of the School of Psychology at Northeast Normal University for human participant research, and each participant or their parents provided informed consent prior to participating in the study.

### Materials and Measures

#### Tendency to Forgive Scale

The Tendency to Forgive Scale (TTF) is a four-item scale which assesses individual differences in the tendency to forgive one’s offense across situations and relationships (Brown, 2003). Sample items include “I tend to get over it quickly when someone hurts my feelings.” The participants were asked to response on a five-point Likert scale that ranges from 1 (strongly disagrees) to 5 (strongly agree). The TTF has been demonstrated to have reasonable internal reliability and a high degree of stability over 8 weeks in a prior study (Brown, 2003). The Chinese version of the TTF was also shown to have a good level of reliability and validity (Jia et al., 2020). In this study, the TTF had acceptable internal consistency.

#### Transgression-Related Interpersonal Motivations Inventory

The Transgression-Related Interpersonal Motivations Inventory (TRIM) is an 18-item self-report questionnaire that measures the motivational changes of victims toward transgressors (McCullough et al., 2006). Three subscales were included in the TRIM: (1) the revenge subscale includes five items that measure the motivation to seek revenge (e.g., “I want him/her to get what he/she deserves”); (2) the avoidance subscale consists of seven items that assess the motivation to avoid the offender (e.g., I would avoid him/her); (3) the benevolence subscale comprises six items that measure the benevolence motivation toward a transgressor (e.g., “Even though his/her actions hurt me, I still have goodwill for him/her”). Each item is scored on a scale of 1–5 from “strongly disagree” to “strongly agree.” Previous studies have demonstrated high internal consistency and reliability of each subscale, and it is also applicable in the youth sample (e.g., McCullough et al., 2000; McCullough and Hoyt, 2002; Nouri et al., 2019). In the present study, Cronbach’s alpha of each subscale was 0.79 for avoidance motivation, 0.83 for revenge motivation, and 0.71 for benevolence motivation.

#### Interpersonal Reactivity Index

Individual differences in empathy were measured using two subscales of the Interpersonal Reactivity Index (Davis, 1980), the empathic concern and the perspective-taking, which were thought to assess affective empathy and cognitive empathy, respectively. The empathic concern subscale is composed of six items and the perspective-taking subscale comprises five items. The response options ranged from 1 (not at all) to 5 (extremely). The responses to these 11 items were averaged to form an empathy index. The Chinese version of the Interpersonal Response Indicator scale has good reliability and validity (Zhang et al., 2010). In the present study, Cronbach’s alpha was 0.73.

### Statistical Analysis

Data analyses were conducted using SPSS Statistics 22.0 and the PROCESS macro for SPSS (Hayes, 2013). An independent-sample *t*-test was used to analyze the possible gender differences in these variables using the current data. Based on our hypothesis, Pearson’s correlations were used to analyze the bivariate correlations between the variables of interest. In the test of the mediating effect of empathy, the bootstrap method in the PROCESS macro for SPSS was used to test the statistical significance of the indirect effects in this study.

## Results

### Descriptive Statistics and Correlations

Table 1 displays the descriptive statistics and the correlations for the study variables as obtained in the current sample of 445 adolescent students. As expected, the participants’ avoidance motivation and revenge motivation were negatively and significantly correlated with their empathy [*r*_(__445__)_ = −0.18, *p* < 0.001; *r*_(__445__)_ = −0.32, *p* < 0.001] and forgiveness scores [*r*_(__445__)_ = −0.35, *p* < 0.001; *r*_(__445__)_ = −0.52, *p* < 0.001]. In contrast, the adolescents’ benevolence motivation is positively and significantly associated with their empathy [*r*_(__445__)_ = 0.33, *p* < 0.001] and forgiveness scores [*r*_(__445__)_ = 0.30, *p* < 0.001]. In addition, a significant positive correlation between empathy and forgiveness among adolescents was also observed [*r*_(__445__)_ = 0.33, *p* < 0.001]. According to three correlation coefficients, we can see that the correlation between revenge motivation and forgiveness is the largest, followed by the correlation between avoidance motivation and forgiveness, and finally the correlation between benevolence motivation and forgiveness is the smallest.

**TABLE 1:** Descriptive statistics and correlations among variables (*N* = 445).

Variables	*M*	SD	1	2	3	4
Forgiveness	13.30	2.69	–	–	–	–
Avoidance	22.34	5.22	−0.35***	–	–	–
Revenge	12.08	4.25	−0.52***	0.56***	–	–
Benevolence	18.25	4.31	0.30***	−0.43***	−0.39***	–
Empathy	2.43	0.59	0.33***	−0.18***	−0.32***	0.33***

***p < 0.001.

### Gender Differences Among Variables

Table 2 shows the gender differences between the variables in the current sample. As shown in the table, avoidance motivation (*t* = −5.944, *p* < 0.001, *d* = 0.571) and benevolence motivation (*t* = 3.757, *p* < 0.001, *d* = 0.369) have significant gender differences, while forgiveness, revenge motivation, and empathy have no significant gender differences. Moreover, boys have higher benevolence motivation and lower avoidance motivation than girls.

**TABLE 2:** Gender differences among variables.

Variables	Gender	*M*	SD	*t*	*d*
Forgiveness	Male	13.50	2.623	1.349	–
	Female	13.15	2.738		
Avoidance	Male	20.69	4.984	−5.944***	0.571
	Female	23.56	5.067		
Revenge	Male	11.80	4.363	–1.203	–
	Female	12.29	4.156		
Benevolence	Male	19.13	4.403	3.757***	0.369
	Female	17.60	4.122		
Empathy	Male	2.432	0.615	0.097	–
	Female	2.427	0.576		

***p < 0.001.

### Mediation Analysis

**FIGURE 1:**
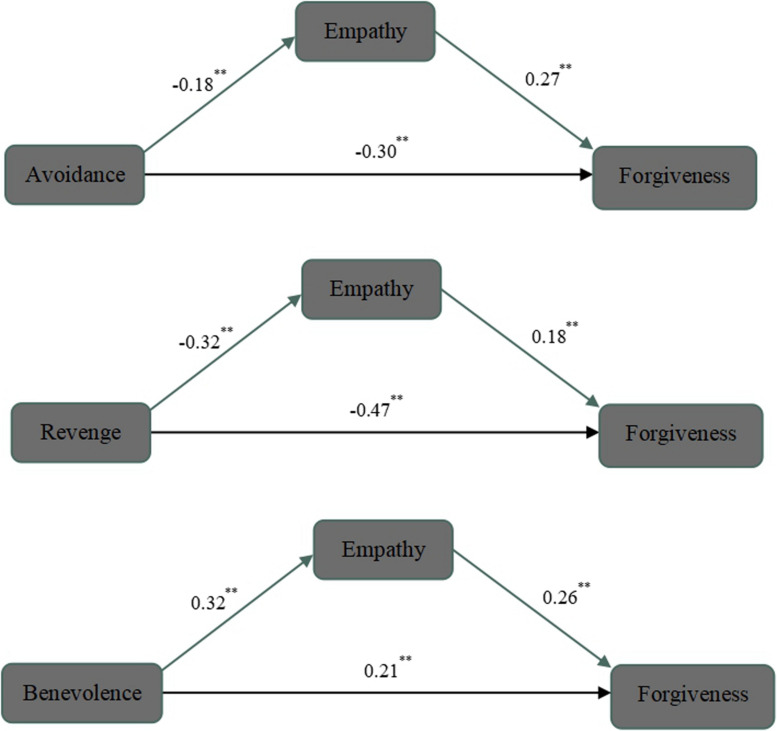
Mediation model.

Based on the results of the correlation analysis, we used model 4 in the PROCESS program to test the mediating effect of empathy between transgression-related interpersonal motivations and the tendency to forgive. All variables’ scores were converted to z-scores first in this analysis. The regression coefficients of each path were significant (see Figure 1). The direct prediction effect of avoidance, revenge, and benevolence motivation on forgiveness tendency was significant. Therefore, these three models were all partial mediating model. Furthermore, bootstrap estimates (based on 5,000 bootstrap samples) indicated that the mediator effects of empathy between avoidance [β = −0.05, CI (−0.09, −0.02)], revenge [β = −0.06, CI (−0.09, −0.03)], and benevolence motivations after transgression [β = 0.08, CI (0.05, 0.13)] and the tendency to forgive were significant. In other words, motivations after aggression not only directly impact adolescents’ forgiveness but also improve the tendency to forgive through the mediating effect of empathy. The 95% confidence intervals of each point estimation are shown in Table 3.

**TABLE 3:** Mediating effects of empathy between motivation and forgiveness.

Model	Effect	SE	Boot LLCI	Boot ULCI	Ratio of total effects (%)
Avoidance–empathy–forgiveness	−0.05	0.02	−0.09	−0.02	14.29
Revenge–empathy–forgiveness	−0.06	0.02	−0.09	−0.03	11.32
Benevolence–empathy–forgiveness	0.08	0.02	0.05	0.13	27.59

## Discussion

The main goal of the current study is to investigate the relationship between motivations after transgression and forgiveness among adolescents and whether empathy mediates the link between transgression-related motivations and forgiveness. Consistent with our hypotheses, the results revealed that the motivation of revenge and avoidance after transgression was negatively correlated with empathy and forgiveness, while the benevolence motivation was positively correlated with empathy and forgiveness. A significant positive correlation between empathy and forgiveness among adolescents was found. More importantly, empathy has a mediating effect between motivations and forgiveness. Moreover, we found that boys showed higher benevolence motivation and lower avoidance motivation than girls.

In the current study, there are significant gender differences in avoidance motivation and benevolence motivation, while there are no gender differences in other variables. This result is partly consistent with previous studies (e.g., Berry et al., 2001; Macaskill et al., 2002; Toussaint and Webb, 2005), and we found that there is no significant gender difference in forgiveness. However, the result of empathy is contrary to most studies (e.g., Macaskill et al., 2002; Toussaint and Webb, 2005; Zhao et al., 2018); we found no gender difference in empathy. The reason may be that the social expectations of gender roles have not significantly affected adolescents, and girls are not as motivated as adult women to understand and care about other people’s thoughts and feelings. The present findings of avoidance and benevolence motivations contradict those of Tang et al. (2012), who showed no apparent gender differences in motivations. The research of Tang et al. takes adults as samples, while the current research takes adolescents as samples. The cognitive level of adolescents being different from adults may explain this contradiction. Adolescent boys are encouraged to be braver than girls, so they choose less to avoid contact with offenders. Moreover, boys and girls have different views on aggressive behavior, which will affect their motivations after aggression (Miller et al., 2008).

The results showed that the low retaliation motivation, avoidance motivation, and high benevolence motivation are associated with forgiveness among adolescents. In line with prior research, teenagers with high forgiveness show less motivation to retaliate, evade after being violated, and are more inclined to show benevolent motivation (Berry et al., 2001). Research found that adolescents tend to avoid after experiencing offenses, which leads to a decrease of self-esteem and long-term emotional reduction, while adolescents who chose forgiveness reported a significant reduction in anger (Watson et al., 2017). Adolescents seem to encounter more interpersonal conflicts, and cognitive, emotional, or behavioral problems will happen if maladaptive coping responses were adopted (Yao and Enright, 2018). Johnson et al. (2013) found that adolescents can solve conflicts successfully, which will contribute to the reorganization and development of youth friendship. In this process, forgiveness plays an important role in restoring interpersonal communication, increasing interpersonal trust, and promoting conflict resolution. Research found that a reduced motivation of retaliation and avoidance can decrease the victims’ anger and behavior problems, which shows the benefits of giving up unforgiveness for mental well-being (Barcaccia et al., 2017). There are differences between different transgression-related motivations and forgiveness, and the correlation between negative motivations and forgiveness is higher, which may be that forgiveness is more promoted by the reduction of negative motivations for adolescents. Barcaccia et al. (2017) have pointed out that teenagers are in an environment of frequent conflicts; forgiving others does not necessarily mean that they increase benevolence and goodwill, but that they only reduce negative motivation in order to maintain an interpersonal relationship.

There is a positive relationship between empathy and forgiveness in adolescents. In addition, empathy is positively correlated with benevolence motivation, while it is negatively associated with avoidance and revenge motivation. This may suggest that the highly empathetic youth tend to focus on others’ experiences in a fairly objective or unselfish manner and more likely to forgive offenders instead of taking revenge and avoidance (Toussaint and Webb, 2005). Christensen et al. (2011) found that empathy is the most salient predictor of forgiveness in the parent–child relationship. A study of forgiveness intervention on female aggressive victims showed that, compared with other groups, victims in the forgiveness intervention group had a significant improvement in empathy and academic performance and a significant reduction in anger, hostility attribution, and delinquent behavior (Park et al., 2013). Moreover, we found the significant mediator effect of empathy between motivations after transgression and forgiveness, namely, aggression-related motivations not only directly affect forgiveness but also indirectly affect forgiveness through empathy. Teenagers with higher empathic ability will concern more about the needs of offenders after being violated (Decety and Yoder, 2016). Empathy may increase the possibility of reconstructing the interpersonal relationship between the victim and the offender, even overriding the harm of the aggression and promoting the occurrence of forgiveness (McCullough et al., 1998; Davis and Gold, 2011). These findings suggested that empathy plays an important role in forgiveness granting among adolescents and support McCullough’s view that empathy is the most salient social cognitive factor in the relationship between aggression-related variables and forgiveness (McCullough et al., 1997).

The current study has several limitations that need to be mentioned. First, self-reported questionnaires were used in the study, which will affect the ecological validity of the results. Subjects may show more empathy and forgiveness because of social expectation effect. Therefore, future research should use more objective ways to measure forgiveness. Second, the current research uses the average score of cognitive and emotional empathy to establish empathy index and does not consider the two dimensions of empathy separately. However, cognitive empathy and emotional empathy are different dimensions of empathy, which may have different mediating effects on the three types of transgression-related motivation and forgiveness. Future studies can explore the mediating effects of cognitive empathy and emotional empathy, respectively. Third, our study is a cross-sectional study, which cannot determine the causal relationship between transgression-related motivations and forgiveness. Thus, longitudinal research can be used to explore how motivations after aggression affect forgiveness in the future.

In general, this study proves that transgression-related motivations can not only directly influence forgiveness but also indirectly affect forgiveness through empathy. The current study extends previous findings concerning transgression-related motivations and forgiveness among Chinese adolescents and provides evidence that empathy plays an important mediating role in the path of motivations and forgiveness. Adolescents are in a period of frequent interpersonal conflicts, so they need to use an adaptive coping style to solve interpersonal conflicts, such as forgiveness. Therefore, it is worthwhile to carry out forgiveness education for adolescents and consider the important role of empathy as well. In school, through forgiveness education, we can improve the empathic ability of adolescents to promote forgiveness and then reduce the negative impact of an interpersonal conflict on them.

## Data Availability Statement

The original contributions presented in the study are included in the article/supplementary material, further inquiries can be directed to the corresponding author.

## Ethics Statement

The studies involving human participants were reviewed and approved by Ethics Committee of Northeast Normal University, China. Written informed consent to participate in this study was provided by the participants’ legal guardian/next of kin.

## Author Contributions

LM and YJ conceived and designed the experiment and contributed to writing the manuscript. LM conducted the experiment and analyzed the data. Both authors contributed to the article and approved the submitted version.

## Conflict of Interest

The authors declare that the research was conducted in the absence of any commercial or financial relationships that could be construed as a potential conflict of interest.
